# Ultra-processed food consumption and adiposity trajectories in a Brazilian cohort of adolescents: ELANA study

**DOI:** 10.1038/s41387-018-0043-z

**Published:** 2018-05-25

**Authors:** Diana Barbosa Cunha, Teresa Helena Macedo da Costa, Gloria Valeria da Veiga, Rosangela Alves Pereira, Rosely Sichieri

**Affiliations:** 1 Department of Epidemiology, Social Medicine Institute, State University of Rio de Janeiro, Rio de Janeiro, Brazil, Rua São Francisco Xavier, 524, 7° andar. Maracanã, Rio de Janeiro, RJ 20550-900 Brazil; 2 Department of Nutrition, University of Brasilia, Campus Darcy Ribeiro, Asa Norte., Brasilia, DF 71910900 Brazil; 3 Department of Nutrition, Federal University of Rio de Janeiro, Av. Carlos Chagas Filho, 373. Edifício do Centro de Ciências da Saúde, Bloco J, 2° andar - Cidade Universitária, Cep, Rio de Janeiro, 21941-590 Brazil

## Abstract

**Background/objectives:**

In Brazil, the increase in obesity rates has been accompanied by increased consumption of ultra-processed food (UPF). The objective of this paper was to evaluate body mass index (BMI) and body fat percentage (%BF) trajectories in adolescents over a 3-year follow-up according to the frequency of UPF consumption.

**Subjects/methods:**

Data of three consecutive years (2010, 2011, and 2012) were obtained from the Adolescent Nutritional Assessment Longitudinal Study (ELANA) that aimed to assess changes in anthropometric indicators of nutritional status, and 1035 adolescents enrolled in the 1st year of high school from six schools (four private and two public) in the metropolitan area of Rio de Janeiro, Brazil were included. At three follow-ups, they had their weights and heights measured. Body composition was measured at the first and second follow-ups. Mixed linear regression models were used to estimate BMI and %BF trajectories based on quartiles of UPF intake, adjusting for type of school, sex, physical activity, and underreporting.

**Results:**

Compared to their counterparts in the 1st quartile, adolescents in the 4th quartile of UPF consumption had a lower daily intake of fruits, cooked vegetables, and raw vegetables and a higher intake of total sugar and physical activity levels (*p* < 0.001). There was an inverse association between UPF consumption and BMI both at baseline and at follow-up. Values for %BF followed the same trend. Adolescents in the 4th quartile had the greatest level of physical activity and lowest total energy intake.

**Conclusion:**

This study confirmed that greater intake of UPF is a marker of an unhealthy diet, but did not support the hypothesis of a high rate of change in BMI associated with greater UPF consumption, even after adjusting for physical activity.

## Introduction

The recommendations of the Brazilian Dietary Guidelines, published in 2014, are based on the degree of processing of food items^1^, and is grounded in the work developed by Monteiro and colleagues^2^, which shows a greater quality of diets with a reduced proportion of ultra-processed foods (UPFs)^2, 3^.

UPFs are defined as formulations modified by industrial procedures using parts or substances extracted from food sources maintaining little or no characteristics of the whole food^2^. During the process, they receive artificial or chemical additives and most of them are energy-dense, composed of low nutrient diversity, but are highly palatable and convenient (ready-to-eat/drink or ready-to-heat), and are heavily advertised^2, 4^. The classification of foods as processed and ultra-processed is recent, and no longitudinal study has previously analyzed the association between the consumption of UPF with weight gain in adolescents. However, cross-sectional studies have described a detrimental association between UPF and dietary quality^2, 5–7^, and also with excessive weight^2, 5, 6, 8–11^.

We have identified only one longitudinal study related to the impact of UPF intake with an increase in serum lipids in Brazilian children, which demonstrated that UPF consumption at 3–4 years of age is a predictor of higher increases in lipoprotein levels profiles at 7–8 years of age^12^.

Adolescence is a period of high nutrient and energy demand and, for this reason, is a nutritionally critical period of life, when lifestyle and dietary habits are changing, making adolescents vulnerable to the consumption of energy-rich and nutrient-poor foods^13, 14^. In Brazil, in the last decade, children and adolescents are the groups with the highest increase in the prevalence of overweight and obese individuals^15^. The hypothesis that the high consumption of UPFs influences weight gain seems plausible, since, in Brazil, the increase in obesity rates has been accompanied by the increased consumption of such foods, as demonstrated in the household budget surveys carried out in the last decade^16^. Thus, the present study aimed to evaluate the association between UPF consumption and adiposity indicator trajectories throughout adolescence.

## Methods

Data were collected from the Adolescent Nutritional Assessment Longitudinal Study (ELANA) cohort, which included adolescents from four private and two public schools located in the metropolitan area of Rio de Janeiro, Brazil, selected based on convenience. All students from the 1st year of high school in 2010 were enrolled and followed up in 2011 and 2012, except pregnant adolescents or adolescents with physical disabilities. The detailed description of the methods of the ELANA study, as well as the sample size calculations and the results of the associations between body mass index (BMI) trajectory and excessive weight gain with demographic and socio-economic factors, were published by Moreira et al^9^.

### Data collection

At baseline, students answered a self-administered questionnaire containing questions regarding lifestyle and dietary behavioral characteristics, and at baseline and at two follow-ups, they had their weights and heights measured. Body composition was measured at baseline and at the first follow-up.

Data on food intake were obtained using a qualitative reduced version of the food frequency questionnaire (FFQ) validated for adolescents of Rio de Janeiro with photograph support by Brito et al^17^. The reduced version of the FFQ used in the ELANA contained 72 food items, with eight consumption frequency options, ranging from “less than once per month or never” to “four or more times a day”. Some items were excluded (based on the frequency of consumption and contribution to the total energy consumption) and others were grouped (e.g., alcohol beverages were combined). The reduced FFQ was also validated against three-day food records and correlation coefficients for nutrients, including calcium, ranged between 0.50 and 0.70, which are considered a good estimation for nutrients.

Anthropometric measures were performed by trained field worker employees (four in each phase of the ELANA) of the Center for Physical Evaluation and Training (CAFT www.caft.com.br). The anthropometric data were collected according to Lohman protocols^18^ and the field workers were standardized according to Habicht techniques^19^ at baseline and according to Norton and Olds^20^ at the three follow-ups. Trained field workers collected anthropometric measures from adolescents who were barefoot and wearing light clothes^18^. Weight was measured using an electronic portable scale with capacity up to 150 kg and variations of 50 g. Height was measured in duplicate using a portable anthropometer with a length up to 200 cm and variations of 0.1 cm. Body composition was measured at baseline and at the second follow-up using a four-pole bioelectrical impedance device (RJL System® 101Q model), validated for children and adolescents^21^. Fat-free mass was estimated using the equation validated by Houtkooper et al^22^. including the resistance values; weight, height, and body fat were estimated based on the difference between total body mass and fat-free mass.

Physical activity was estimated using the Brazilian version^23^ of the short version of the International Physical Activity Questionnaire (IPAQ), which contains questions regarding the frequency and duration of walking, and moderate and vigorous physical activity during the previous week. The classification of the levels of intensity (moderate or vigorous physical activity) was calculated based on the “Guidelines for Data Processing and Analysis of the International Physical Activity Questionnaire (IPAQ)–Short and Long Forms.”^24^

### Data analysis

For analysis, the eight frequency options of food intake were transformed into daily frequency. For energy and nutrient intake estimation, frequencies were multiplied by the portion reported in the original FFQ^25^ and the food composition table of Brazilian foods and dishes^26^ was used.

Foods were classified according to the nature and level of processing^4^. The UPF consumption was estimated by summing up the following items: instant pasta, sweet pastries, cookies, cheese rolls, hot dogs, fried filled rolls, oven-baked filled rolls, pizza, French fries, hamburgers, nuggets, ham, sausages, candies, chocolates, chocolate milk beverages, condensed milk, peanut candies, ice cream, jelly, chips, marmalade, sodas, “guaraná” refreshments, and other sugar-sweetened beverages.

Markers of diet quality (fiber, fruits and vegetables, and total sugar intake) were compared by quartiles of UPF intake, as well as total energy intake.

For the assessment of BMI and body fat percentage (%BF) trajectory, mixed linear regression models were used. These models accounted for the correlations among measures and follow-up losses^27^. Models used the Proc Mixed procedure in the Statistical Analysis System, version 9.4 (SAS Institute Inc, Cary, NC). The time variable in the model was age in months, accounting for the expected BMI variation due to the growth. BMI and %BF trajectories by quartiles of UPF intake were tested by *p*-values < 0.05 of the interaction of UPF * time. Models were adjusted for type of school (public or private), sex, physical activity levels, and underreporting. To account for underreporting, a dummy variable for energy intake below the 10th percentile was included in the analysis.

### Code availability

SAS code is available by the authors upon request.

## Results

Of the 1039 adolescents evaluated at baseline, four did not answer the FFQ. Therefore, the analysis at baseline included 1035 adolescents: 509 from public schools and 526 from private schools (Table 1). The mean age of the participants was 16 years (standard deviation (SD) = 0.9) and 46% were boys (Table 2). Table 2 also shows the participants at each follow-up and their characteristics.

**Table 1:** Mean and standard deviation (SD) of daily frequency of intake (times/day) of foods included in the ultra-processed food group according to school type and sex

Schools' type	Public (*n* = 509)					Private (*n* = 526)				
Food items	Boys (*n* = 225)		Girls (*n* = 284)		*p* value*	Boys *n* = 255		Girls (*n* = 271)		*p* value*
	Mean	SD	Mean	SD		Mean	SD	Mean	SD	
Instant pasta	0.38	0.73	0.31	0.56	0.180	0.17	0.30	0.22	0.47	0.288
Sweet pastry	0.48	0.88	0.42	0.81	0.184	0.18	0.43	0.15	0.33	0.723
Cookies	0.91	1.05	1.01	1.18	0.285	0.69	0.94	0.68	0.95	0.985
Cheese rolls	0.16	0.46	0.19	0.55	0.192	0.12	0.26	0.14	0.40	0.573
Hot dog	0.24	0.65	0.21	0.53	0.733	0.12	0.18	0.11	0.25	0.025
Fried filled rolls	0.38	0.67	0.37	0.72	0.309	0.25	0.34	0.20	0.39	0.018
Oven filled rolls	0.39	0.63	0.42	0.70	0.706	0.33	0.47	0.29	0.43	0.167
Pizza	0.25	0.55	0.22	0.55	0.990	0.18	0.37	0.15	0.23	0.608
French fries	0.49	0.88	0.59	0.95	0.172	0.35	0.60	0.31	0.57	0.058
Hamburger	0.34	0.73	0.30	0.69	0.136	0.20	0.33	0.12	0.17	<0.001
Nuggets	0.26	0.59	0.28	0.68	0.837	0.25	0.56	0.21	0.43	0.630
Ham	0.69	1.01	0.65	0.93	0.919	0.50	0.71	0.38	0.56	0.005
Sausages	0.31	0.58	0.37	0.68	0.418	0.18	0.29	0.18	0.32	0.394
Fruit drinks	1.26	1.33	1.43	1.43	0.150	0.85	1.08	1.06	1.24	0.240
Packed fruit juices	0.78	1.11	0.97	1.32	0.402	0.56	0.91	0.59	0.92	0.658
Soda type “guaraná’’	0.90	1.21	1.00	1.26	0.146	0.67	0.93	0.57	0.92	0.015
Sodas	1.29	1.39	1.13	1.32	0.112	0.98	1.17	0.84	1.19	0.003
Candies	1.01	1.26	1.60	1.51	<0.001	0.93	1.28	1.29	1.45	0.002
Chocolate	0.45	0.85	0.75	1.13	<0.001	0.47	0.74	0.54	0.83	0.537
Chocolate milk beverages	0.94	1.09	1.07	1.18	0.213	0.97	1.01	0.74	0.89	0.002
Condensed milk	0.22	0.49	0.33	0.70	0.001	0.19	0.32	0.20	0.43	0.969
Peanut candy	0.26	0.64	0.30	0.77	0.447	0.15	0.39	0.10	0.38	<0.001
Ice cream	0.35	0.69	0.36	0.78	0.511	0.22	0.36	0.24	0.44	0.604
Jelly	0.18	0.37	0.30	0.71	0.270	0.13	0.32	0.14	0.38	0.944
Chips	0.39	0.69	0.52	0.81	0.002	0.30	0.49	0.29	0.50	0.449
Marmalade (“doce de fruta”)	0.26	0.69	0.17	0.57	0.022	0.12	0.34	0.08	0.26	0.064
Ultra-processed foods (items/day)	13.02	11.25	14.66	12.36	0.075	9.80	6.82	9.43	7.01	0.419

* Comparison preformed with Wilcoxon rank scores test

**Table 2:** Agec, anthropometry, and prevalence of overweight and obesity of student´s participant in each follow-up wave

	1st year				2nd year				3rd year			
	(*n* = 1035)				(*n* = 787)				(*n* = 585)			
	Mean	SD	range		Mean	SD	range		Mean	SD	range	
Age (year)	15.7	0.9	13.5	19.5	16.6	0.8	14.4	19.8	17.6	0.8	15.3	20.9
Weight (kg)	61.2	13.7	30.5	126.9	63.4	14.0	32.3	137.2	65.4	15.2	35.7	130.6
Height (m)	1.7	0.1	1.4	2.0	1.7	0.1	1.4	2.0	1.7	0.1	1.4	2.0
BMI (kg/m2)	22.0	4.1	14.5	40.0	22.6	4.2	14.8	40.5	23.1	4.2	15.4	41.3
Prevalence overweight (%)	18.6				20.3				19.8			
Prevalence obesity (%)	8.9				8.2				8.3			

The 25 UPFs analyzed in this study and respective daily frequencies of consumption according to sex and school type are shown in Table 1. Adolescents in the public schools reported greater daily frequency of UPF consumption at baseline (mean ± SD of items consumed by adolescents in public school = 14.5 ± 12.5; in private schools = 9.94 ± 7.2, *p* < 0.001). There were differences in the frequency of intake of specific UPFs between boys and girls in public and private schools. Hot dogs, fried rolls, ham, hamburgers, and sweet peanuts were eaten more frequently by boys than by girls. Sugar-sweetened beverages (guaraná), chocolate milk beverages, and soft drinks were also consumed more frequently by boys than by girls in private schools. On the other hand, boys from public schools ate marmalade more frequently than girls did and girls from public schools consumed condensed milk, chocolate, and chips more frequently than boys did. Candies were more frequently consumed by girls than by boys in both schools (Table 1).

During the 3-year follow-up, weight and BMI increased. There were losses during follow-up and the adolescents in the final follow-up presented prevalence rates of being overweight of 19.8% and obese of 8.3% (Table 2).

Energy, total fiber density, and sugar density increased from the 1st to the 3rd quartile of UPF intake and decreased in the 4th quartile with a positive significant trend. Density intake decreased for raw and cooked vegetables and fruits, supporting a decrease in the quality of diet with increasing intake of UPFs. Additionally, more teenagers were classified as moderately or vigorously active in the 4th quartile compared with the 1st. Trends by quartiles were all significant, except for the 2nd follow-up BMI. Compared to their counterparts in the 1st quartile, adolescents in the 4th quartile of UPF consumption had a lower daily intake of fruits (257 vs. 339.7 g/kcal), cooked vegetables (20.9 vs. 30.1 g/kcal), and raw vegetables (31.2 vs. 41.5 g/kcal), and a higher intake of total sugar (319.6 vs. 234.2 g/kcal), higher levels of physical activity at baseline, and lower BMI at all follow-ups (Table 3).

**Table 3:** Total energy, density of total fiber and select food groups, body mass index (BMI), and body fat (% BF) according to quartiles of ultra-processed foods (UPF) consumed by adolescents

	1st quartile of UPF		2nd quartile of UPF		3rd quartile of UPF		4th quartile of UPF		*P* value*
Energy and foods items	Mean	SD	Mean	SD	Mean	SD	Mean	SD	
Total Energy (kcal/day)	3761	2943	4351	3596	4969	4068	3701	2109	<0.001
Total Fiber (g/Kcal)	32.5	26.7	38.6	32.7	41.6	34.0	30.6	19.5	<0.001
Total Sugar (g/Kcal)	234.2	194.7	295.4	235.0	355.9	269.8	319.6	181.3	<0.001
Fruits (g/Kcal)	339.7	526.4	412.4	658.9	383.7	613.6	257.0	420.5	<0.001
Cooked vegetables (g/Kcal)	30.1	65.4	34.5	66.5	32.1	73.2	20.9	45.3	<0.001
Raw vegetables (g/Kcal)	41.5	64.1	54.8	86.3	42.8	74.1	31.2	46.2	<0.001
BMI (kg/m^2^)									
Baseline	23.1	4.6	22.2	4.3	21.7	3.8	21.3	3.5	<0.001
1st follow-up	23.5	4.6	22.8	4.2	22.4	4.1	21.8	3.6	<0.001
2nd follow-up	24.0	4.3	23.0	4.5	23.0	4.3	22.4	3.7	0.40
Body fat (%)									
Baseline	25.1	10.1	23.8	9.9	23.7	8.0	21.9	8.6	0.088
2nd follow-up	25.9	8.8	23.3	8.2	23.5	8.3	22.1	7.9	<0.001
Physical activity	(%)		(%)		(%)		(%)		
Active by WHO classification at baseline	17.0		23.7		23.0		25.8		<0.001

* *P* value for trend

At baseline, boys with normal weight presented a daily frequency intake of UPFs (mean ± SD) and daily energy intake of 12.5 ± 10.3 g and 4321 kcal, respectively, compared to 9.7 ± 7.7 g and 3350 kcal, respectively, for those who were overweight or obese. Among girls, these values were 13.1 ± 11.0 g and 4345 kcal, respectively, for those with normal weight and 10.9 ± 10.7 g and 3853 kcal, respectively, for those who were overweight or obese (data not shown). Since the associations are quite similar by sex, the final analysis combined both sexes.

**Fig. 1:**
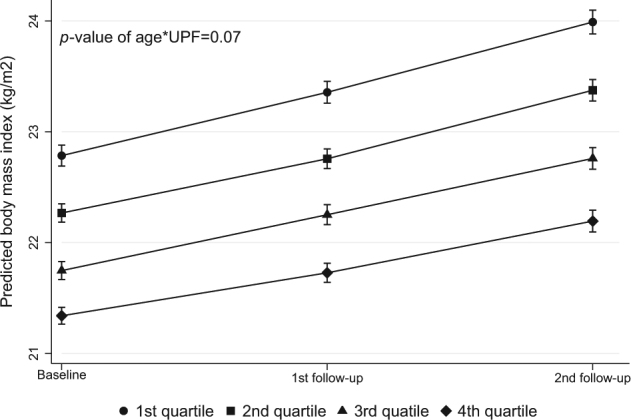
Predicted BMI values during follow-up for Brazilian adolescents students according to quartiles of ultra-processed food frequency intake. Rio de Janeiro, Brazil 2010−2012

At baseline, there was a clear inverse association between BMI and intake of UPFs, and values of predicted BMI during the follow-up period showed no interaction (*p* = 0.07) between age and quartiles of UPF consumption (Fig. 1) with a slightly higher increase in BMI among the students in the 1st quartile of UPF intake. Values for %BF followed the same trend.

Underreporting of intake (coefficient = −0.94, *p* = 0.04) and physical activity (coefficient = −1.0, *p* < 0.001) were statistically associated with BMI at baseline and at follow-up. Analysis including these variables, and further adjustment for total energy intake materially changed the results. The same occurred for the exclusion of those who were obese at baseline (data not shown).

## Discussion

Adolescents in the 4th quartile of UPF consumption at baseline had lower BMI and %BF and an increasing rate over three years of follow-up than adolescents classified in the 1st quartile. Thus, the study did not support the hypothesis that high intake of UPFs is associated with greater weight gain in adolescents.

Many possibilities were explored that could explain these unexpected findings. Firstly, obese children at baseline were excluded from analysis since they could be avoiding energy-dense foods to reduce weight, but this was not the case. The inclusion of underreporting, a characteristic more frequent among overweight and obese individuals, also did not change the results. Moreover, the use of an FFQ made UPF identification difficult, since it is based on a fixed list of foods and food groups, many of which should be classified as ultra-processed or unprocessed foods, depending on the method of cooking (e.g., lasagna, hamburger).

In agreement with the results of other studies^5, 28^, UPF intake was associated with an unhealthy dietary profile since the consumption of vegetables, fruits, and fiber decreased with an increase of UPF intake, while total sugar intake increased. However, energy intake increased from the 1st to the 3rd quartile and showed an important reduction in the 4th quartile. These data may suggest that those adolescents with the highest UPF consumption more frequently exhibited normal weight and also exercised more than those in the other quartiles.

Our results are in line with those of cross-sectional studies that have shown an inverse association between energy intake and BMI both in adults^29^ and children^30^. In both studies, controlling for underreporting changed the direction of the association. We attempted to adjust for underreporting by including a variable marker for those below the 10th percentile of energy intake. This is suboptimal because underreporting should be defined as a relationship between energy intake and energy expenditure, but FFQ does not allow for this comparison. Adjusting for energy intake also did not change our results.

UPF intake is a marker for an unhealthy diet; however, experimental studies aimed at displacing high-energy foods by increasing the consumption of vegetables and fruits were not successful ^31^ and systematic reviews have not confirmed an increase in the intake of fruits as an adequate intervention for the prevention of obesity^32^. These findings suggest that obesity is much more a problem of quantity than of quality of food consumption.

Specifically among adolescents, a meta-analysis involving fruits and vegetables delivered at home did not change weight gain in experimental studies^32^. Although eating different foods can have a greater or lower contribution in total energy intake, every intake contributes to energy. Thus, data from a nationwide Brazilian survey comparing days with and without consumption of specific items showed that only the days with intake of vegetables decreased the total energy intake. For many other items, such as fruit, milk, and beans, energy intake was greater on the day the item was reported, compared with the day that it was not reported, for the same individuals^33^. This is the great challenge of treating and preventing obesity because there is no replacement of one type of food for another. As an example, the acquisition of unhealthy food groups in Brazil is associated with greater diversity of the diet, but great diversity is positively associated with excess weight gain and inversely associated with being underweight^34^. In short, healthy and unhealthy food groups are combined in diet and obesity prevention requires messages focused on eating less.

A limitation of this study is the lack of data on food intake in the follow-ups to test whether changes in UPF consumption are related to changes in BMI. This was the design used in the Nurse study that showed many specific foods related to weight gain in adults^35^. However, those individuals who change their intake during follow-up may do so because of a health outcome, mainly to avoid weight gain. Although IPAQ could not accurately capture physical activity data, we observed that compared to their counterparts in the 1st quartile, adolescents in the 4th quartile of UPF consumption had higher physical activity levels.

In conclusion, the results favor the hypothesis that to curb excessive weight gain, messages regarding the quality of diet should be combined with a reduction in food intake.

## References

[1] Ministry of Health of Brazil. Dietary Guidelines for the Brazilian Population. Ministry of Health of Brazil, Brazil, 2014.

[2] Monteiro CA, Levy RB, Claro RM, de Castro IR, Cannon G (2011). Increasing consumption of ultra-processed foods and likely impact on human health: evidence from Brazil. Public Health Nutr..

[3] Monteiro CA (2009). Nutrition and health. The issue is not food, nor nutrients, so much as processing. Public Health Nutr..

[4] Monteiro CA, Levy RB, Claro RM, Castro IR, Cannon G (2010). A new classification of foods based on the extent and purpose of their processing. Cad. Saude Publica.

[5] Bielemann RM, Motta JV, Minten GC, Horta BL, Gigante DP (2015). Consumption of ultra-processed foods and their impact on the diet of young adults. Rev. Saude Publica.

[6] Luiten CM, Steenhuis IH, Eyles H, Ni Mhurchu C, Waterlander WE (2015). Ultra-processed foods have the worst nutrient profile, yet they are the most available packaged products in a sample of New Zealand supermarkets. Public Health Nutr..

[7] Moubarac JC, Martins AP, Claro RM, Levy RB, Cannon G, Monteiro CA (2012). Consumption of ultra-processed foods and likely impact on human health. Evidence from Canada. Public Health Nutr..

[8] Canella DS, Levy RB, Martins AP, Claro RM, Moubarac JC, Baraldi LG (2014). Ultra-processed food products and obesity in Brazilian households (2008-2009). PLoS ONE.

[9] Moreira NF, Sichieri R, Reichenheim ME, Oliveira AS, Veiga GV (2015). The associations of BMI trajectory and excessive weight gain with demographic and socio-economic factors: the Adolescent Nutritional Assessment Longitudinal Study cohort. Br. J. Nutr..

[10] OPAS. (2015). Ultra-processed food and drink products in Latin America: Trends, impact on obesity, policy implications.

[11] Tavares LF, Fonseca SC, Garcia Rosa ML, Yokoo EM (2011). Relationship between ultra-processed foods and metabolic syndrome in adolescents from a Brazilian Family Doctor Program. Public Health Nutr..

[12] Rauber F, Campagnolo PD, Hoffman DJ, Vitolo MR (2014). Consumption of ultra-processed food products and its effects on children’s lipid profiles: a longitudinal study. Nutr. Metab. Cardiovasc Dis..

[13] Hobbs M, Pearson N, Foster PJ, Biddle SJ (2015). Sedentary behaviour and diet across the lifespan: an updated systematic review. Br. J. Sports Med..

[14] Lobstein T, Jackson-Leach R, Moodie ML, Hall KD, Gortmaker SL, Swinburn BA (2015). Child and adolescent obesity: part of a bigger picture. Lancet.

[15] Niehues JR, Gonzales AI, Lemos RR, Bezerra PP, Haas P (2014). Prevalence of overweight and obesity in children and adolescents from the age range of 2 to 19 years old in Brazil. Int J. Pediatr..

[16] Martins AP, Levy RB, Claro RM, Moubarac JC, Monteiro CA (2013). Increased contribution of ultra-processed food products in the Brazilian diet (1987-2009). Rev. Saude Publica.

[17] Brito AP, Araujo MC, Guimaraes CP, Pereira RA (2017). Validade relativa de questionário de frequência alimentar com suporte de imagens. Ciência e Saúde Coletiva.

[18] Lohman TG, Roche A, Martorell R (1988). Anthropometric Standardization Reference Manual..

[19] Habicht JP (1974). Estandarizacion de metodos epidemiológicos cuantitativos sobre el terreno (Standardization of quantitative epidemiological methods in the field. Bol. Oficina Sanit. Panam..

[20] Pederson D, Gore C, Norton K, Olds T (1996). Anthropometry measurement error. Antropometrica.

[21] Guo SM, Roche AF, Houtkooper L (1989). Fat-free mass in children and young adults predicted from bioelectric impedance and anthropometric variables. Am. J. Clin. Nutr..

[22] Houtkooper LB, Going SB, Lohman TG, Roche AF, Van Loan M (1992). Bioelectrical impedance estimation of fat-free body mass in children and youth: a cross-validation study. J. Appl. Physiol. (1985).

[23] Guedes DP, Lopes CC, Guedes JERP (2005). Reprodutibilidade e validade do Questionário Internacional de Atividade Física em adolescentes. Rev. Bras. Med. Esport..

[24] Guidelines for data processing and analysis of the International Physical Activity Questionnaire (IPAQ)—Short and Long Forms. IPAQ. https://www.ipaq.ki.se/dloads/IPAQ%20LS%20Scoring%20Protocols_Nov05.pdf. Published November 2005.

[25] Araujo MC, Yokoo EM, Pereira RA (2008). Validation and calibration of a semiquantitative food frequency questionnaire designed for adolescents. J. Am. Diet. Assoc..

[26] Instituto Brasileiro de Geografia e Estatística (IBGE). Pesquisa de orçamentos Familiares. Tabela de Composição Nutricional dos Alimentos Consumidos no Brasil. Rio de Janeiro: IBGE; 2011. https://www.ibge.gov.br/home/estatistica/populacao/condicaodevida/pof/2008_2009_composicao_nutricional/pofcomposicao.pdf.

[27] Fitzmaurice GM, Laird NM, Ware JH (2011). Applied longitudinal analysis..

[28] Louzada ML, Martins AP, Canella DS, Baraldi LG, Levy RB, Claro RM (2015). Impact of ultra-processed foods on micronutrient content in the Brazilian diet. Rev. Saude Publica.

[29] Mendez MA, Popkin BM, Buckland G, Schroder H, Amiano P, Barricarte A (2011). Alternative methods of accounting for underreporting and overreporting when measuring dietary intake-obesity relations. Am. J. Epidemiol..

[30] Bornhorst C, Huybrechts I, Hebestreit A, Vanaelst B, Molnar D, Bel-Serrat S (2012). Diet-obesity associations in children: approaches to counteract attenuation caused by misreporting. Public Health Nutr..

[31] Bayer O, Nehring I, Bolte G, von Kries R (2013). Fruit and vegetable consumption and BMI change in primary school-age children: a cohort study. Eur. J. Clin. Nutr..

[32] Kaiser KA, Brown AW, Bohan Brown MM, Shikany JM, Mattes RD, Allison DB (2014). Increased fruit and vegetable intake has no discernible effect on weight loss: a systematic review and meta-analysis. Am. J. Clin. Nutr..

[33] Sichieri R, Bezerra IN, Araujo MC, de Moura Souza A, Yokoo EM, Pereira RA (2015). Major food sources contributing to energy intake—a nationwide survey of Brazilians aged 10 years and older. Br. J. Nutr..

[34] Bezerra IN, Sichieri R (2011). Household food diversity and nutritional status among adults in Brazil. Int J. Behav. Nutr. Phys. Act..

[35] Mozaffarian D, Hao T, Rimm EB, Willett WC, Hu FB (2011). Changes in diet and lifestyle and long-term weight gain in women and men. N. Engl. J. Med..

